# Comparative-genomic analysis reveals dynamic NLR gene loss and gain across Apiaceae species

**DOI:** 10.3389/fgene.2023.1141194

**Published:** 2023-03-02

**Authors:** Xiaohui Liang, Junming Dong

**Affiliations:** The State Key Laboratory of Pharmaceutical Biotechnology, School of Life Sciences, Nanjing University, Nanjing, Jiangsu, China

**Keywords:** Apiaceae, NLR gene, plant disease resistance, evolutionary pattern, gene family

## Abstract

**Introduction:** Nucleotide-binding leucine-rich repeat (NLR) genes play a crucial role in green plants’ responding to various pathogens. Genome-scale evolutionary studies of NLR genes are important for discovering and applying functional NLR genes. However, little is known about the evolution of NLR genes in the Apiaceae family including agricultural and medical plants.

**Methods:** In this study, comparative genomic analysis was performed in four Apiaceae species to trace the dynamic evolutionary patterns of NLR genes during speciation in this family.

**Results:** The results revealed different number of NLR genes in these four Apiaceae species, namely, Angelica sinensis (95), Coriandrum sativum (183), Apium graveolens (153) and Daucus carota (149). Phylogenetic analysis demonstrated that NLR genes in these four species were derived from 183 ancestral NLR lineages and experienced different levels of gene-loss and gain events. The contraction pattern of the ancestral NLR lineages was discovered during the evolution of D. carota, whereas a different pattern of contraction after first expansion of NLR genes was observed for *A. sinensis*, *C. sativum* and *A. graveolens*.

**Discussion:** Taken together, rapid and dynamic gene content variation has shaped evolutionary history of NLR genes in Apiaceae species.

## 1 Introduction

To cope with the invasion of various pathogenic microorganisms, plants have evolved two layers of immune systems to protect themselves (Wang et al., 2020; Zhou and Zhang, 2020). Relying on the pattern recognition receptors on the surface of plant cells, the first layer immune system helps the plants to recognize conserved pathogen-associated molecular patterns (PAMPs) of bacterial cells, namely, PAMP-triggered immunity (PTI) (Bigeard et al., 2015). Correspondingly, pathogens can block PTI signaling by releasing effector proteins into plant cells (Bigeard et al., 2015). In response to the blocking of bacterial effectors, plants have evolved the second layer immune system that detects the presence of these effectors *via* intracellular disease resistance genes (R genes) and further induces downstream immunity, namely, effector-triggered immunity (ETI) (Dangl et al., 2013; Wang et al., 2020). Since the first R gene was cloned and characterized in the late 19th century, more than 300 functional R genes have been identified (Johal and Briggs, 1992; Kourelis and van der Hoorn, 2018).

Currently, over 60% of R genes belong to a family that encodes nucleotide-binding site (NBS) and leucine-rich-repeat (LRR) domain receptors, well known as NBS-LRR or NLR genes (Kourelis and van der Hoorn, 2018). Based on the different N-terminal structural domains, NLR genes were classified into three subclasses, including TNL containing the TIR structural domain, CNL with the CC structural domain, and RNL characterized by the RPW8 structural domain (Whitham et al., 1994; Parker et al., 1997; Botella et al., 1998; Pan et al., 2000; Shao et al., 2014). The majority of CNL and TNL proteins function as detectors of pathogens’ effectors (Kourelis and van der Hoorn, 2018), while RNL proteins typically act as “helper” NLRs and get involved in the downstream signaling of CNL and TNL proteins (Kourelis and van der Hoorn, 2018; Wang et al., 2020).

The development of sequencing technology has generated plenty of plant genomes. The genome-scale identification of NLR gene composition not only helps to reveal the evolutionary patterns of NLR gene families in plants, but is also crucial for discovering and applying functional NLR genes, especially for agricultural or industrial crops. Liu et al. (2021) revealed that the convergent contraction of NLR genes was associated with ecological adaptability and NLR subclasses co-evolved with plant immune pathway components. Several studies at the family level have revealed distinctive evolutionary patterns of different plant taxa, such as NLR gene contraction of Poaceae species (Luo et al., 2012), consistent expansion of NLR genes in Fabaceae species (Jia et al., 2015) and first expansion and then contraction of NLR genes in Brassicaceae species (Zhang et al., 2016). Besides, genome-scale NLR gene analysis of multiple wheat lines led to successful cloning of the insect resistance gene *Sm1* (Walkowiak et al., 2020). A highly conserved NLR gene, *Pb3*, was screened based on genome-wide association analysis of 230 rice germplasm genomes and rice blast resistance phenotypes, and further molecular experiments demonstrated that Pb3 could confer rice blast resistance (Ma et al., 2022).

Apiaceae species are annual or perennial herbs that are widely distributed in temperate regions of the world (Fox et al., 2022). Recently, the genomes of seven species from Apiaceae, including Apioideae and Mackinlayoideae, have been sequenced (https://www.plabipd.de/plant_genomes_pa.ep). Apiaceae species have always played an important role in socio-economic and human life, among which *Angelica sinensis*, *Bupleurum chinense, Centella asiatica* and *O. javanica* are medicinal plants (Lu and Li, 2019; Sun et al., 2020; Liu et al., 2022), as well as *Apium graveolens*, *Coriandrum sativum* and *D. carota* are vegetables (Fox et al., 2022). Previous studies have shown that the Ks peak of the paralogous genes in five Apioideae species, including *Daucus carota*, *Oenanthe javanica*, *A*. *sinensis*, *C*. *sativum* and *A*. *graveolens*, corresponded to a recent WGD (whole genome duplication) event, possibly specific in Apioideae instead of Mackinlayoideae (Han et al., 2022). A variety of pathogens are known to be capable of infecting Apiaceae plants and causing economic losses (Fox et al., 2022). However, few studies reported the composition and evolutionary pattern of NLR genes in the Apiaceae family, limiting the discovery of functional NLR genes and their application in production. In this study, the four Apiaceae species from different genera, with fully annotated genomic data, were collected and used to decipher the evolutionary features of NLR genes in this family.

## 2 Materials and methods

### 2.1 Identification and classification of NLR genes

Genomic sequences of four Apiaceae species were downloaded from the available database (Table 1). Detailed information related to the genomes is shown in Table 1. Three Apiaceae species were excluded due to the lack of the gff3 annotation files, namely, *B. chinense*, *O. javanica* and *C. asiatica*. The identification of NLR genes was performed as described previously (Shao et al., 2014). In brief, the NBS (named as NB-ARC) domain was identified using both hidden Markov models search (HMMsearch) and BLAST methods. The HMM profile of the NBS domain (Pfam no. PF00931) was downloaded from the Pfam database and used as a query to search for NLR proteins from four Apiaceae species *via* hmmer3.3 (Johnson et al., 2010) with the E-value setting to 10^–4^. To avoid missing possible candidates, the resulting protein sequences were further used to run a BLASTp search against all protein sequences in each genome (E-value = 1.0). To verify whether all hits indeed possessed the NBS domain, they were then subjected to hmmscan analysis using hmmer3.3 (Johnson et al., 2010) against a local Pfam-A database with the E-value setting to 10^–4^. MEME analysis was conducted to annotate conserved motifs in the NBS domain of all the identified NLR genes (Bailey et al., 2009). Conserved motifs were visualized using WebLogo (Crooks et al., 2004).

**TABLE 1:** Metadata of the genomes from four Apiaceae species.

Species	References	Download-link
*Angelica sinensis*	Han et al. (2022). The Plant Journal	https://data.cyverse.org/dav-anon/iplant/home/licheng_caas/Angelica.sinensis_genome/
*Apium graveolens*	Cheng et al. (2022). iScience	https://ngdc.cncb.ac.cn/search/?dbId=gwh&q=GWHBGBL00000000+
*Coriandrum sativum*	Song et al. (2020). The Plant Journal	http://cgdb.bio2db.com/Download/
*Daucus carota*	Iorizzo et al. (2016). Nature Genetics	https://www.ncbi.nlm.nih.gov/assembly/GCF_001625215.1

### 2.2 Chromosomal distributions of NLR genes

Chromosomal distribution patterns of NLR genes were identified as described previously (Ameline-Torregrosa et al., 2008). In brief, the genomic positions of obtained NLR genes were extracted from the gff3 annotation files. Then the sliding-window analysis was performed according to the 250 kb window size. An NLR gene was regarded as a singleton locus if no other NLR genes were found upstream or downstream of 250 kb region flanking it. Comparatively, if the distance between two annotated NLR genes was less than 250 kb, they were considered as a clustered locus. Besides, syntenic relationships of NLR genes were analyzed and visualized using Tbtools (Chen et al., 2020).

### 2.3 Phylogenetic analysis of NLR genes

The amino acid sequences of the NBS domains were extracted from all the identified NLR genes. Multiple sequence alignment was performed using ClustalW with default parameters, and then artificially adjusted with MGEA X (Kumar et al., 2018). Those sequences with too short length were removed from the aligned results. The resulting alignments were subjected to the phylogenetic analysis using IQ-TREE with the maximum likelihood method (Minh et al., 2020). The best-fit model of nucleotide substitution was selected by ModelFinder (Kalyaanamoorthy et al., 2017). Branch support values were estimated using SH-aLRT (Anisimova et al., 2011) and UFBoot2 (Minh et al., 2013) with 1,000 bootstrap replicates, and the resulting files were visualized and annotated with iTOL (Letunic and Bork, 2021).

### 2.4 Gene loss/duplication events of NLR genes

The comparative analysis between phylogenetic tree of NLR genes and species tree was performed using Notung software to determine gene loss/duplication events of NLR genes (Stolzer et al., 2012). The MCScanX package was used to analyze the types of NLR gene duplication in a given genome according to a pair-wise all-against-all blast of protein sequences (Wang et al., 2012).

## 3 Results

### 3.1 Compositions of NLR genes in four Apiaceae species

In this study, the number of NLR genes in the investigated four Apiaceae species showed some variations with ranging from 95 in *A. sinensis* to 183 in *C. sativum* (Table 2). Taken as a reference, the number of NLR genes in the other three species was 1.95 times (*C. sativum*), 1.61 times (*A. graveolens*) and 1.57 times (*D. carota*) as many as that of *A. sinensis*, respectively (Table 2). The subclass classification of NLR genes showed that each of four Apiaceae species possessed all the three subclasses, namely, CNL, TNL and RNL, proposed by previous study (Shao et al., 2016) (Table 2). Among these three NLR subclasses, CNL genes accounted for the highest percentage for each species, reaching 92.62% (*D. carota*), 63.93% (*C. sativum*), 57.52% (*A. graveolens*) and 55.79% (*A. sinensis*), respectively (Table 2). Generally, all Apiaceae species have more CNL and TNL genes than RNL genes, except for *D. carota* which also possessed a small number of TNL genes (Table 2).

**TABLE 2:** The number and composition of identified NLR genes in the studied four Apiaceae species.

NLR composition	*A. sinensis*	*C. sativum*	*A. graveolens*	*D. carota*
NLR	95	183	153	149
CNL	53 (55.79%)	117 (63.93%)	88 (57.52%)	138 (92.62%)
TNL	39 (41.05%)	59 (32.24%)	59 (38.56%)	6 (4.03%)
RNL	3 (3.16%)	7 (3.83%)	6 (3.92%)	5 (3.35%)
Clustered loci	38 (41.76%)	82 (55.03%)	47 (46.08%)	98 (65.77%)
Singleton loci	53 (58.24%)	67 (44.97%)	55 (53.92%)	51 (34.23%)
Unmapped	4	34	51	0

**FIGURE 1:**
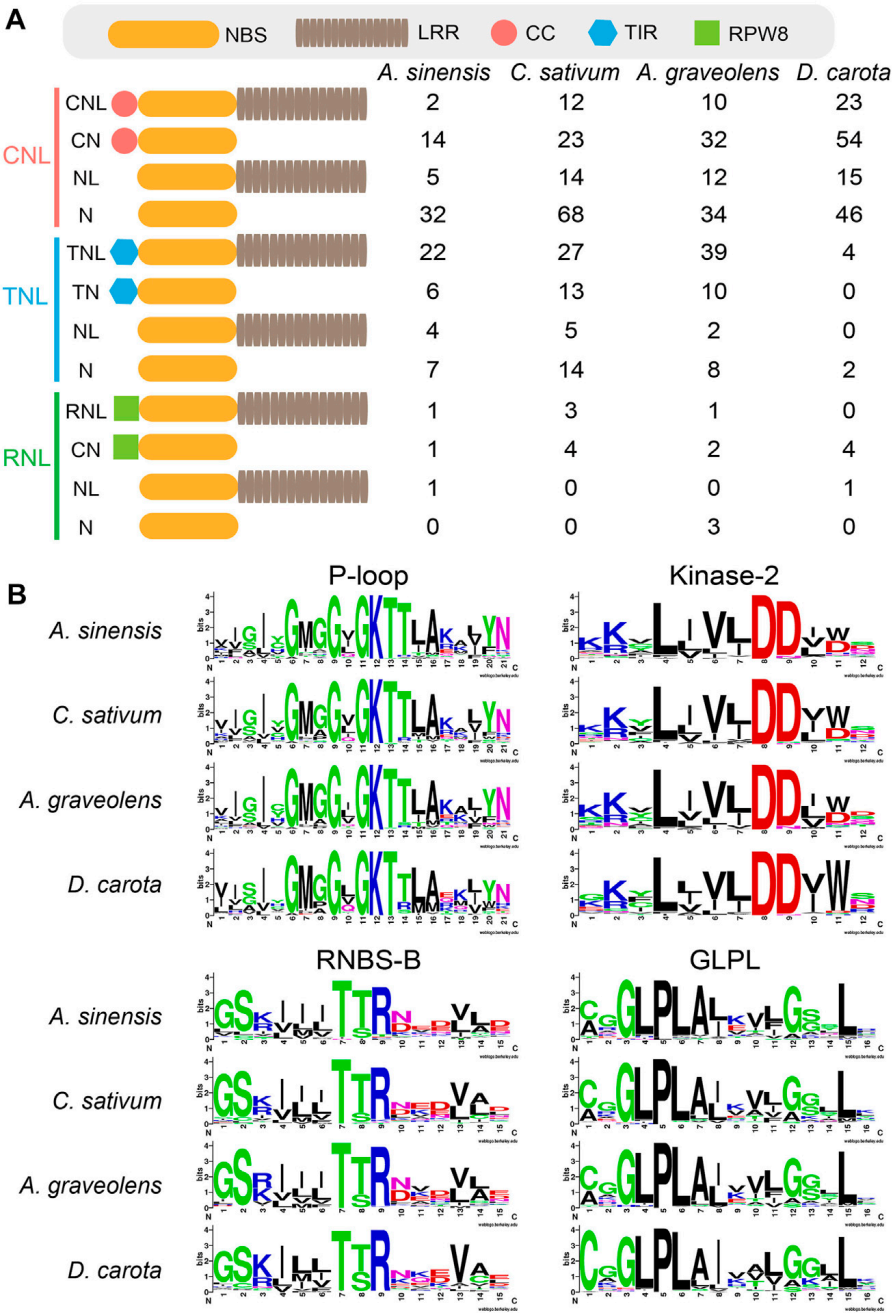
Domain structures and key motifs of NLR genes in four Apiaceae species. **(A)** Domain compositions and arrangements of NLR proteins encoded by the identified NLR genes from four Apiaceae species. C, CC domain; T, TIR domain; R, RPW8 domain; N, NBS domain; L, LRR domain. **(B)** Four key motifs in the conserved NBS domains from four Apiaceae species.

Domain analysis showed high structure diversity of NLR proteins among four Apiaceae species (Figure 1). The identified NLR proteins could be divided into 12 groups based on their domain composition and arrangement (Figure 1A). Among them, only most TNL genes could encode complete TNL proteins containing the N-terminal TIR domain, the central NBS domain and the C-terminal LRR domain (Figure 1A). However, most CNL genes could encode incomplete CNL proteins lacking the N-terminal CC domain, or the C- terminal LRR domain, or both (Figure 1A). A similar scenario was also observed for most RNL genes. The trends in four Apiaceae species were overall consistent. For MEME analysis, four key motifs, namely, P-loop, Kinase-2, RNBS-B and GLPL, were detected in the amino acid sequences of NBS domains from four Apiaceae species (Figure 1B). These four key motifs were found to be conserved in NLR proteins of four Apiaceae species (Figure 1B), as reported in other angiosperms (Shao et al., 2016).

### 3.2 Chromosomal distribution patterns of NLR genes in four Apiaceae species

**FIGURE 2:**
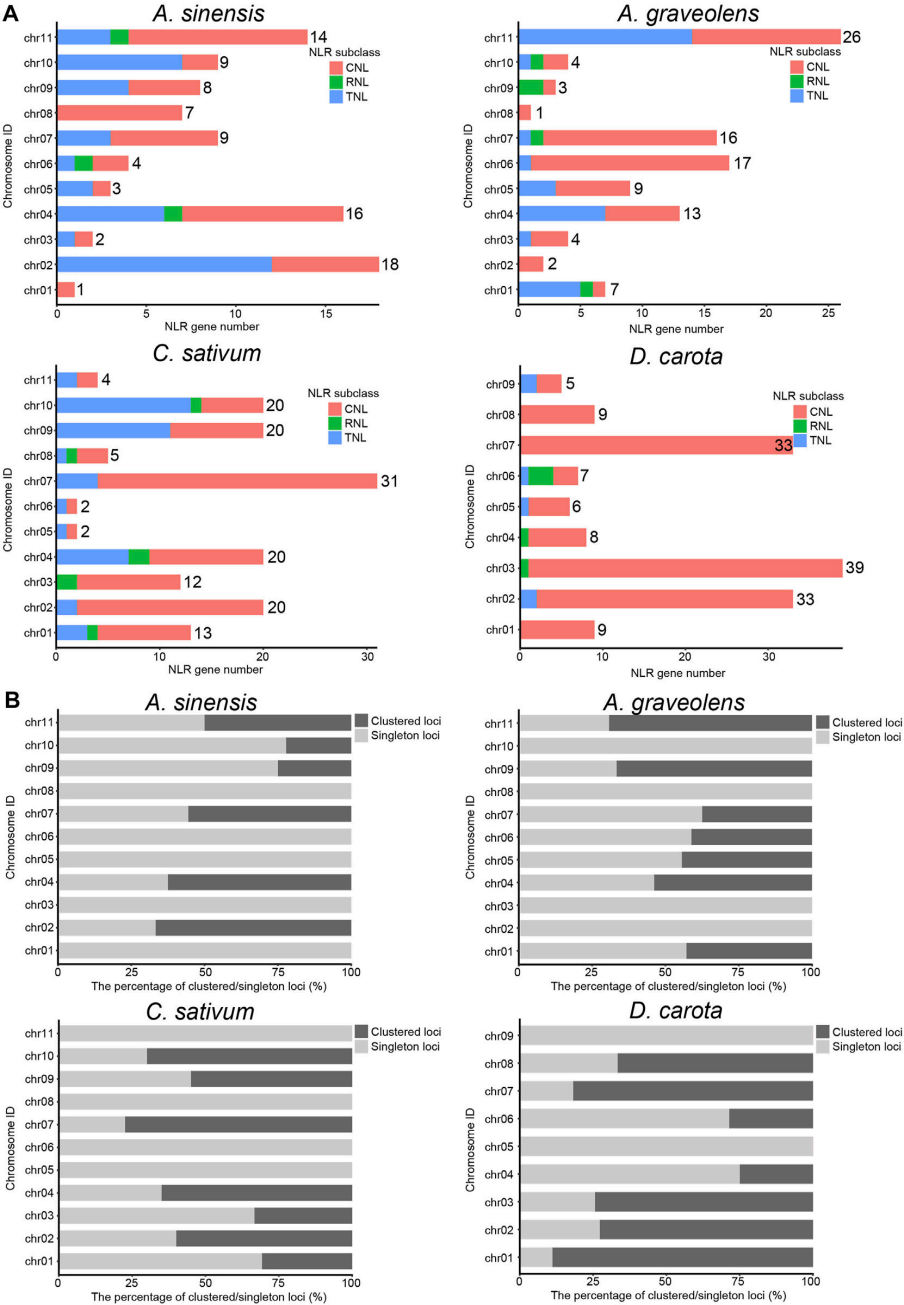
The composition and chromosomal distribution of NLR genes in four Apiaceae species. **(A)** The number variation of NLR genes among different genomes from four Apiaceae species. Three NLR subclasses are indicated with different colors, with red for CNL, green for RNL and blue for TNL. The number of NLR genes on each chromosome is shown. **(B)** The percentage of clustered/singleton loci among different genomes from four Apiaceae species.

To explore the clustering organization, we further analyzed the chromosomal distribution patterns of NLR genes in the four Apiaceae species. The results demonstrated that the majority of NLR genes could be mapped to the specific chromosomes for each species (Figure 2A), except for four (4.21%) in *A. sinensis*, 34 (18.58%) in *C. sativum*, 51 (33.33%) in *A. graveolens* and zero (0%) in *D. carota* due to that these NLR genes were annotated only at the scaffold level (Table 2). Besides, an uneven gene distribution was found among different chromosomes in each species (Figure 2A). When the chromosome with a minimum number of NLR genes was used as a reference, the times of NLR gene on other chromosomes ranged from two to 18 in *A. sinensis*, from two to 15 in *C. sativum*, from two to 26 in *A. graveolens*, and from one to six in *D. carota* (Figure 2A).

As to the clustering situations, the majority of NLR genes belonged to clustered loci rather than singleton loci in two species, namely, *C. sativum* with 55.03% and *D. carota* with 65.77% (Table 2). However, the number of NLR genes treated as singleton loci was slightly more than that of NLR genes classified as clustered loci in the remaining two species (Table 2). Besides, the number of clustered loci showed different trends among species, with the highest number in *D. carota* (98), followed by *C. sativum* (82), *A. graveolens* (47) and *A. sinensis* (38) (Table 2). When considering the chromosomal scale, clustered loci were found in almost all chromosomes with the high number of NLR genes (Figure 2A) and the percentage of clustered loci showed some differences among different chromosome for each of these four Apiaceae species, ranging from 22.22% to 66.67% in *A. sinensis*, 33.33%–77.42% in *C. sativum*, 37.50%–69.23% in *A. graveolens* and 25.00%–88.89% in *D. carota* (Figure 2B).

**FIGURE 3:**
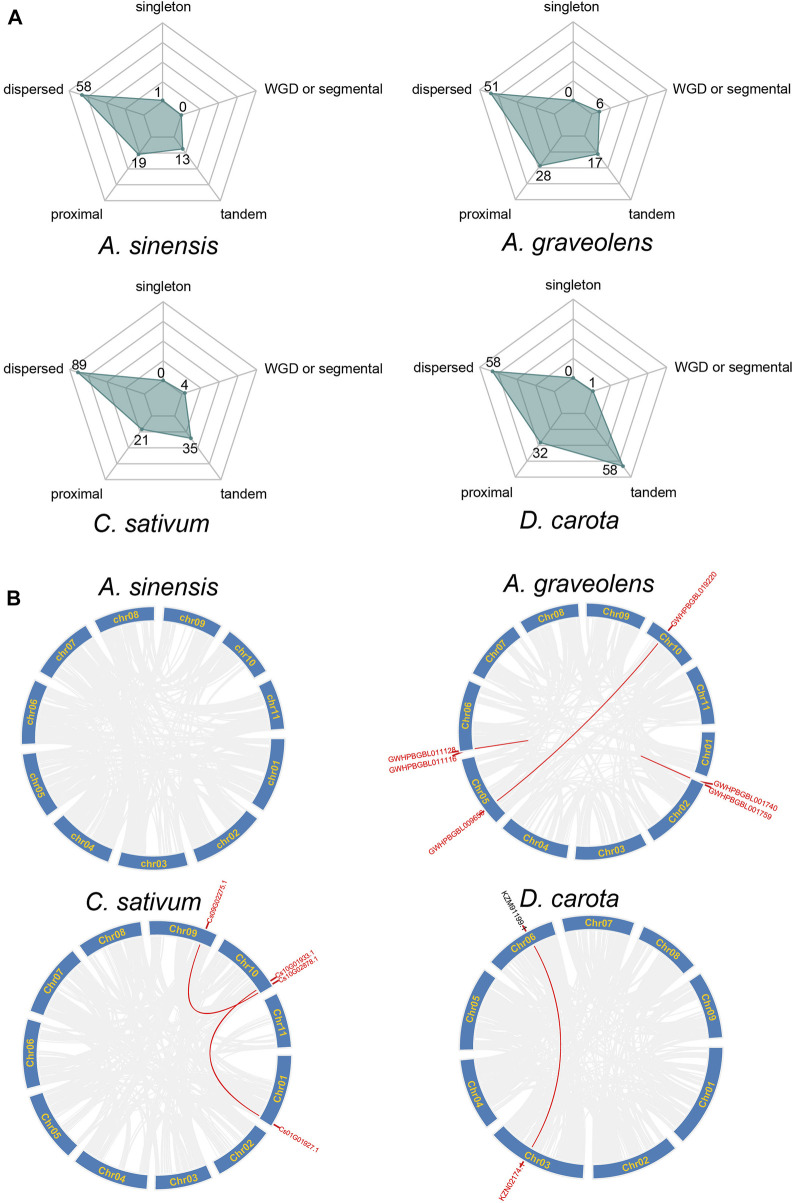
Duplication types of NLR genes across Apiaceae species. **(A)** Type of NLR gene duplication in *A. sinensis*, *A. graveolens*, *C. sativum* and *Daucus carota*, respectively. The number of NLR genes derived from each type was shown. **(B)** Syntenic relationships of NLR genes in *A. sinensis*, *A. graveolens*, *C. sativum* and *Daucus carota*, respectively. Gene ID in red represents NLR genes and gene ID in dark belongs to non-NLR gene.

The emergence of clustered loci of NLR genes may be mainly attributed to the occurrence of duplication events (Leister, 2004). Therefore, the duplication types of NLR genes were further investigated in these four Apiaceae species. The results showed that the expansion of NLR genes was dominated by different duplication types among species (Figure 3A). Most of the NLR genes in *A. sinensis* (63.74%, 58/91), *C. sativum* (59.73%, 89/149), *A. graveolens* (50%, 51/102) and *D. carota* (38.93%, 58/149) were resulted from dispersed duplication, whereas the moderate percentage of NLR genes in these four Apiaceae species were characterized as tandem/proximal duplications, except that the NLR gene number *via* tandem duplication events was consistent with that derived by dispersed duplication in *D. carota* (Figure 3A). Only a small percentage of NLR genes in *A. graveolens* (5.88%, 6/102), *C. sativum* (2.68%, 4/149) and *D. carota* (0.67%, 1/149) were generated by whole genome duplications (WGD)/segmental duplication, whereas no WGD/segmental duplicated NLR genes were found in *A. sinensis* (Figure 3A). Correspondingly, the syntenic relationships among NLR genes, implying possible WGD events, were discovered in *A. graveolens*, *C. sativum* and *D. carota*, and no syntenic NLR gene was identified in *A. sinensis* (Figure 3B). Notably, the WGD or segmental duplication event was detected between a RNL gene (KZN02174.1) and a gene (KZM91199.1) only encoding RPW8 domain in *D. carota* (Figure 3B).

### 3.3 Phylogenetic analysis of NLR genes

**FIGURE 4:**
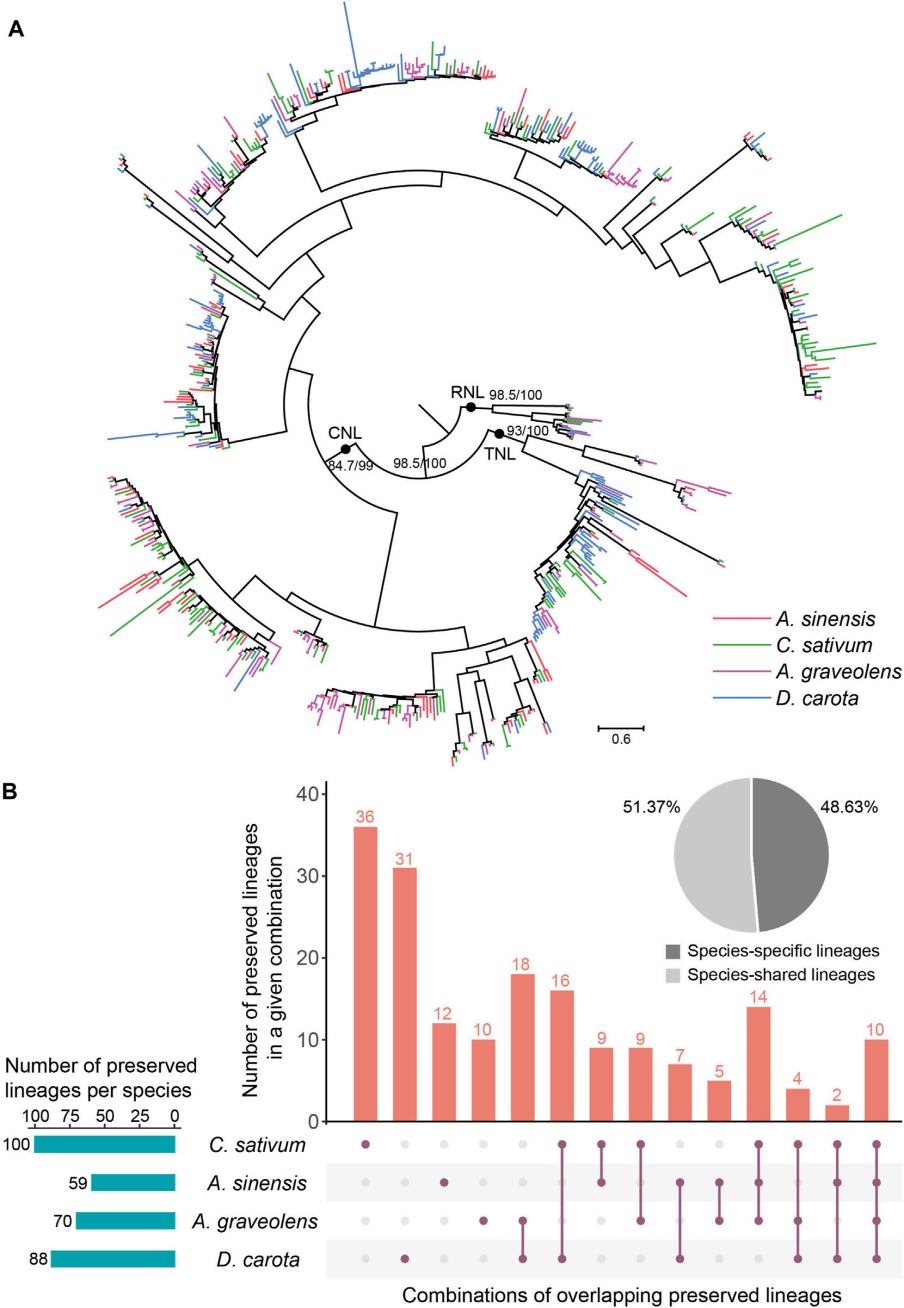
Phylogenetic and evolutionary analysis of NLR genes in four Apiaceae species. **(A)** The phylogeny was reconstructed based on the conserved NBS domain of NLR genes in the four Apiaceae species. Branch support values are obtained using SH-aLRT (%) and UFBoot2 (%), and are labeled on basal nodes. The scale bar represents 0.6 substitutions per site. **(B)** Specie-shared and species-specific lineages were derived from the 183 ancestral NLR lineages. The total number of preserved lineages per species were shown. The combination, *A. sinensis*, *A. graveolens* and *Daucus carota*, was not displayed due to the lack of the situation that no lineages were found to be preserved in *A. sinensis*, *A. graveolens* and *Daucus carota*, and only lost in *C. sativum*.

To trace the evolutionary history of NLR genes in Apiaceae, their phylogenetic relationships were constructed based on the amino acid sequences of the conversed NBS domain (Figure 4A). The results showed that NLR genes of the studied four Apiaceae species formed three distinct clades with high support values (>80), corresponding to CNL, TNL and RNL subclasses, respectively (Figure 4A). Compared to the CNL and TNL clades, the branch lengths of the RNL clade were relatively shorter (Figure 4A), suggesting that RNL genes had a slower evolutionary rate. In addition, within the CNL and TNL clades, clustering situations of CNL genes and/or TNL genes from a single species were frequently observed (Figure 4A), possibly attributed to species-specific expansions of CNL and/or TNL genes.

To understand the evolutionary patterns of NLR genes during the speciation of these four Apiaceae species, comparative analysis of species tree and gene tree based on NBS domain was performed using Notung software (Stolzer et al., 2012). The results revealed that the common ancestor of these four Apiaceae species possessed at least 183 ancestral NLR lineages, including 143 CNL, 32 TNL and eight RNL (Supplementary Figure S1). These ancestral NLR lineages were further termed as “Apiaceae NLR lineages”. At the lineage level, species-specific and ancestral loss events were found in these four Apiaceae species (Figure 4B). By assigning the NLR genes in each species to the 183 ancestral Apiaceae NLR lineages, the results showed that 59, 100, 70 and 88 Apiaceae NLR lineages were inherited by *A. sinensis*, *C. sativum*, *A. graveolens* and *D. carota*, respectively (Figure 4B). Among these ancestral NLR lineages, only 10 of them were preserved in all the four genomes (Supplementary Table S1). In contrast, 89 lineages were species-specific (*A. sinensis*, 12; *C. sativum*, 36; *A. graveolens*, 10; *D. carota*, 31), reaching 48.63% of the Apiaceae NLR lineages (Figure 4B). The remaining 94 lineages were differentially preserved in two to three species, accounting for 51.37% of the Apiaceae NLR lineages (Figure 4B). Notably, no lineages were found to be preserved in *A. sinensis*, *A. graveolens* and *D. carota*, and only lost in *C. sativum*.

### 3.4 Evolutionary trajectory of NLR genes in Apiaceae species

**FIGURE 5:**
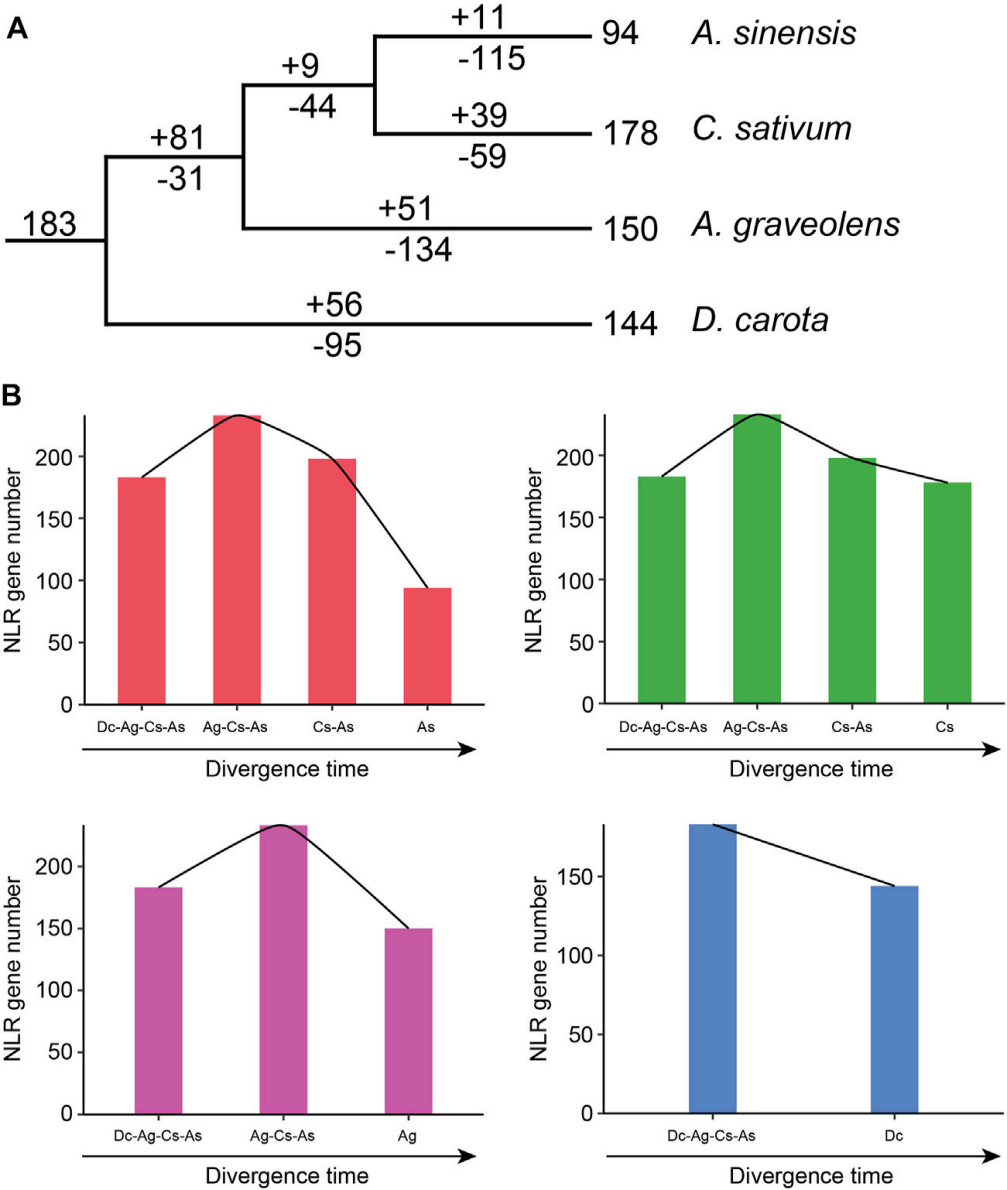
Evolutionary trajectory of NLR genes during Apiaceae species speciation. **(A)** Number variations of NLR genes at different stages of four Apiaceae species. Differential gene-gain and loss events are indicated by numbers with + or - on each branch. **(B)** Dynamic evolutionary patterns of NLR genes at different stages of four Apiaceae species. As, *A. sinensis*; Cs, *C. sativum*; Ag, *A. graveolens*; Dc, *Daucus carota*.

Based on the loss and gain events of NLR genes in each divergence node of Apiaceae species, the evolutionary trajectories of NLR genes in these four Apiaceae species were traced. The 183 ancestral Apiaceae NLR lineages were identified in the common ancestor of these four Apiaceae species (Figure 5A). After separation from the common ancestor of *A. graveolens*, *C. sativum* and *A. sinensis* (Ag-Cs-As node), 95 gene-loss and 56 gain events were detected during the evolution of *D. carota* (Figure 5A), suggesting a pattern of NLR contraction during this period (Figure 5B). However, a different pattern of “first expansion and then contraction” of NLR genes was found for the remaining three species, namely, *A. sinensis* (As), *C. sativum* (Cs) and *A. graveolens* (Ag) (Figure 5B). The expansion events were only detected during the period from the common ancestor of these four Apiaceae species to the Ag-Cs-As node, with 81 gene-gain and only 31 gene-loss events (Figure 5A). After the above-mentioned expansion events, the successive contraction pattern was discovered during the period that the Ag-Cs-As node further diverged into *A. graveolens* (Ag), *C. sativum* (Cs) and *A. sinensis* (As) (Figure 5B). Although different gene-loss and gene-gain events were both found, the number of gene-loss events was more than that of gene-gain events during each evolutionary period since the Ag-Cs-As node (Figure 5A). After separation from the common ancestor of *C. sativum* and *A. sinensis* (Cs-As node), 44 gene-loss and only nine gain events were detected during the evolution of *A. graveolens* (Figure 5A), suggesting a contraction pattern of NLR genes (Figure 5B). The Cs -As node then diverged to generate *A. sinensis* and *C. sativum*. In *A. sinensis*, 115 gene-loss and only 11 gain events were discovered, leading to the existence of 94 NLR genes (Figure 5A). Using this strategy, a similar pattern of “consistent contraction” was also observed for NLR genes in *C. sativum*, with 59 gene-loss and 39 gene-gain events (Figure 5A).

## 4 Discussion

Shao et al. (2019) suggest that NLR genes originated prior to the last common ancestor of green plants, due to the presence of NLR genes in all the lineages of green plants, as well as the absence in Rhodophyta and Glaucophyta. The NLR genes are well known as a large gene family in angiosperms, with an average of several hundred members per species (Liu et al., 2021). Systematic genome-wide studies of NLR genes have greatly contributed to discovering and applying functional NLR genes in important crops and economic plants in recent years (Walkowiak et al., 2020; Ma et al., 2022). However, the lack of informative evolution on NLR genes has heavily hampered identification and application of functional NLR genes in Apiaceae species. Taking advantage of available genomic data from the four Apiaceae species, the evolutionary profile of NLR genes was analyzed from multiple perspectives in this study, which can provide scientific guidance for breeding of the Apiaceae species.

The intensive genome-scale studies have shown that the number of NLR genes varied dramatically across different species in recent years (Xue et al., 2020; Liu et al., 2021; Santos et al., 2022). For example, both *Apostasia shenzhenica* and *Phalaenopsis equestris* have less than 100 NLR genes (Xue et al., 2020), whereas barley possesses 468 NLR genes (Li et al., 2021) and *Coffea eugenioides* has more than 1000 NLR genes (Santos et al., 2022). In this study, a total of 95, 183, 153 and 149 NLR genes were identified in *A. sinensis*, *C. sativum*, *A. graveolens* and *D. carota*, respectively (Table 2). Among all investigated angiosperms up to now, the Apiaceae species were slightly poor in NBS genes, compared to the average of 300 NLR genes in angiosperms (Liu et al., 2021). However, some differences were detected for the number of NLR genes among these four Apiaceae species. The genomes of *C. sativum*, *A. graveolens* and *D. carota* had more than 1.5 times the number of NLR genes as *A. sinensis*, suggesting that species-specific gene-loss and gain events occurred after their separation from the common ancestor. Even if a similar number of NLR genes was found between *A. graveolens* and *D. carota*, significantly different compositions of NLR genes were identified among them (Table 2). This might be attributed to the differential inheritance of ancestral NLR lineages from the common ancestor in these two species (Figure 4B, Figure 5A).

Reconstructing the ancestral states of NLR genes showed that the common ancestor of these four Apiaceae species likely possessed at least 183 ancestral NLR lineages, including 143 CNL, 32 TNL and eight RNL (Supplementary Figure S1). The number of ancestral NLR lineages in Apiaceae is larger than that in Fabaceae (119, Shao et al., 2014) and Solanaceae (176, Qian et al., 2017), and slightly fewer than that in Brassicaceae (228, Zhang et al., 2016). Besides, the number of ancestral NLR lineages in Apiaceae is much larger than the ancestral NLR lineage number in several monocot groups, such as 30 ancestral lineages in orchids (Xue et al., 2020) and 101 ancestral NLR lineages in Arecaceae (Li et al., 2021). The above-mentioned dicot families had larger ancestral NLR lineage numbers than that in several monocot families, attributed in part to their preserved an additional NLR subclass, TNL. Notably, 10 ancestral NLR lineages were found readily preserved in these four Apiaceae species (Figure 4B), representing four different genera and inhabiting different environments. Those genes in each species, belonging to the 10 ancestral NLR lineages, were available in Supplementary Table S1 and could be potential candidates for the discovery and experimental validation of functional NLR genes. Besides, the absence of orthologous genes in closely related species within the same family may indicate loss of plant defense against specific pathogens. On this regard, specific probes/markers can be designed to validate the association of presence/absence of NLR genes with plant resistant to various pathogens of Apiaceae species in future studies.

The number of NLR genes in plants has undergone a rapid and dynamic evolution through frequent gene-gain and gene-loss events in response to the fluctuations of external pathogenic environment (Shao et al., 2019; Liu et al., 2021). For example, the Cucurbitaceae and Poaceae species experienced a similar contraction pattern of NLR genes (Li et al., 2010; Luo et al., 2012; Wan et al., 2013), whereas those species in Fabaceae and Rosaceae exhibited a different pattern of NLR gene “consistent expansion” (Shao et al., 2014; Jia et al., 2015). In this study, two different evolutionary modes of NLR genes were revealed in these four Apiaceae species after their diverging from the common ancestor. A distinct “first expansion and then contraction” pattern of NLR genes was observed in the three Apiaceae species (Figure 5), consistent with previous reports in the five Brassicaceae species (Zhang et al., 2016), and some degree of contraction pattern was discovered for *D. carota* (Figure 5).

## 5 Conclusion

This study systematically analyzed the profiles of NLR genes in four Apiaceae species and revealed their compositions and evolutionary modes, which might provide a resource for discovering and applying functional NLR genes. Phylogenetic analysis showed that NLR genes in these four Apiaceae species were derived from 183 ancestral lineages, and two distinct patterns, “first expansion and then contraction” and “consistent contraction”, were observed for NLR genes in these four species. Species-specific lineage loss and gene duplication have shaped the current NLR profiles of different Apiaceae species. The obtained results can serve as a valuable resource for genetic breeding of Apiaceae species.

## Data Availability

The original contributions presented in the study are included in the article/Supplementary Material, further inquiries can be directed to the corresponding author.
